# Humanization of the mouse mammary gland by replacement of the luminal layer with genetically-engineered preneoplastic human cells

**DOI:** 10.1186/s13058-014-0504-9

**Published:** 2014-12-20

**Authors:** Stephanie Verbeke, Elodie Richard, Elodie Monceau, Xenia Schmidt, Benoit Rousseau, Valerie Velasco, David Bernard, Herve Bonnefoi, Gaetan MacGrogan, Richard D Iggo

**Affiliations:** 1 INSERM U916, Bergonié Cancer Institute, University of Bordeaux, Bordeaux, France; 2 Bute Medical School, University of St Andrews, Bordeaux, France; 3 Animalerie A2, University of Bordeaux, Bordeaux, France; 4 Pathology Department, Bergonié Cancer Institute, Bordeaux, France; 5 INSERM U1052, Centre Leon Berard, University of Lyon, Lyon, France; 6 Postal address: Institut Bergonié, 229 cours de l’Argonne, Bordeaux, 33076 France

## Abstract

**Introduction:**

The cell of origin for estrogen receptor α (ERα) positive breast cancer is probably a luminal stem cell in the terminal duct lobular units. To model these cells we have used the murine myoepithelial layer in the mouse mammary ducts as a scaffold upon which to build a human luminal layer. To prevent squamous metaplasia, a common artifact in genetically engineered breast cancer models, we sought to limit activation of the epidermal growth factor receptor (EGFR) during *in vitro* cell culture before grafting the cells.

**Methods:**

Human reduction mammoplasty cells were grown *in vitro* in WIT medium. Epidermal growth factor (EGF) in the medium was replaced with amphiregulin and neuregulin to decrease activation of EGFR and increase activation of EGFR homologs 3 and 4 (ERBB3 and ERBB4). Lentiviral vectors were used to express oncogenic transgenes and fluorescent proteins. Human mammary epithelial cells were mixed with irradiated mouse fibroblasts and matrigel, then injected through the nipple into the mammary ducts of immunodeficient mice. Engrafted cells were visualized by stereomicroscopy for fluorescent proteins and characterized by histology and immunohistochemistry.

**Results:**

Growth of normal mammary epithelial cells in conditions favoring ERBB3/4 signaling prevented squamous metaplasia *in vitro*. Normal human cells were quickly lost after intraductal injection but cells infected with lentiviruses expressing *CCND1*, *MYC*, *TERT*, *BMI1* and a short hairpin RNA targeting *TP53* were able to engraft and progressively replace the luminal layer in the mouse mammary ducts, resulting in the formation of an extensive network of humanized ducts. Despite expressing multiple oncogenes, the human cells formed a morphologically normal luminal layer. Expression of a single additional oncogene, *PIK3CA*-*H1047R*, converted the cells into invasive cancer cells. The resulting tumors were ERα+, Ki67+ luminal B adenocarcinomas that were resistant to treatment with fulvestrant.

**Conclusions:**

Injection of preneoplastic human mammary epithelial cells into the mammary ducts of immunodeficient mice leads to replacement of the murine luminal layer with morphologically normal human cells. Genetic manipulation of the injected cells makes it possible to study defined steps in the transformation of human mammary epithelial cells in a more physiological environment than has hitherto been possible.

**Electronic supplementary material:**

The online version of this article (doi:10.1186/s13058-014-0504-9) contains supplementary material, which is available to authorized users.

## Introduction

Pioneering studies by the Weinberg group have shown that normal human cells can be fully converted into tumor cells by targeting a small number of processes known to be abnormal in cancer, including telomere maintenance, the restriction point, cell cycle checkpoints and apoptosis [1]. Human mammary epithelial cells transformed in this way have been widely used but frequently form tumors with histological features rarely seen in human breast cancer, in particular squamous carcinoma [2]. This is probably caused by excessive activation of EGFR [3]. Since squamous metaplasia increases with time in culture, current approaches attempt to shorten the transformation process as much as possible, often to as little as 24 hours [4],[5]. Squamous changes are also commonly seen in transgenic mouse models of breast cancer, where part of the explanation may lie in mistargeting of oncogenes to myoepithelial or bipotent progenitor/stem cells rather than luminal stem cells [6].

About 70% of human breast cancers express the estrogen receptor alpha (ERα, *ESR1*), but genetically defined tumors based on transformation of normal human cells are usually ERα negative. Since the *ESR1* gene is rarely amplified in breast cancer, ERα expression is normally ascribed to a lineage choice that traps the cells in an ERα + state. The most likely cell of origin for this event is a luminal progenitor or stem cell located in the terminal duct-lobular unit (TDLU) [7].

Traditional breast cancer models based on injection of tumor cells directly into the mammary fat pad [8] or under the renal capsule [9] do not take into account the unique features of the microenvironment in which breast cancers develop. Behbod and colleagues recently described an intraductal injection technique that disseminates tumor cells throughout the mouse mammary ductal tree, including the TDLUs [10]. This approach places potential tumor cells at or near the normal point of origin of breast cancer and faithfully reproduces the histology of human ductal carcinoma in situ (DCIS) [11].

We describe an approach based on the Behbod intraductal injection technique using genetically engineered cells cultured in conditions favoring ERBB3 signaling. Cells transduced with vectors expressing *CCND1*, *MYC*, *TERT*, *BMI1* and a short hairpin RNA targeting p53 (*TP53*) were able to form a morphologically normal luminal layer. Introduction of a single additional activated oncogene, *PIK3CA-H1047R*, converted them into ERα + invasive ductal adenocarcinomas.

## Methods

### Lentiviral vectors

The lentiviral plasmid vector expressing *TERT* and puromycin acetyltransferase (*pac*) (pSD-83) was described by Duss et al [12]. The vector expressing *BMI1* and *CFP* (pER7) was constructed from pSD-84 [12] by replacing the *pac* gene with *CFP* by standard cloning. The vector expressing *MYC* and hygromycin phosphohydrolase (*hph*) (pSV32) was constructed by shuffling the *MYC* open reading frame from pSD-94 [12] into pLenti PGK-hygro DEST (Addgene 19066) by Gateway cloning. The vector expressing *CCND1* and neomycin phosphotransferase (*npt*) (pSV31) was constructed by Gateway cloning of the *CCND1* open reading frame from pENTR-CCND1 (PlasmID HsCD00001252) into pLenti PGK-Neo DEST (Addgene 19067). The pLVTH-sip53 vector expressing *GFP* and a short hairpin RNA targeting *TP53* was obtained from Addgene (12239). The vector expressing *PIK3CA-H1047R* and *hph* (pER157) was constructed by cloning the *PIK3CA-H1047R* open reading frame from pBABE-PIK3CA (PlasmID clone 25920) into pENTR1A to give pER152, then transferred by Gateway cloning into pLenti CMV/TO-hygro DEST (Addgene 17484). The vector expressing tdTomato (pER5) was kindly provided by Francois Moreau-Gaudry.

### Western blot analysis

Cells were collected four to seven days post-transfection and lysed in sodium dodecyl sulfate polyacrylamide gel electrophoresis (SDS-PAGE) sample buffer [13]. Cell lysates were separated by SDS-PAGE and transferred onto nitrocellulose membranes. The membranes were blocked with 5% fat-free milk powder in 150 mM NaCl, 50 mM Tris-HCL pH 8.0, 0.1% Tween-20 (TBST) and incubated overnight at 4°C with the following antibodies: TERT (Y182), PI3K p110α (04-399), ΔN-p63 (p40 [5]-[17] PC373), GATA3 (09-076) (Millipore); BMI1 (D20B7), keratin 18 (DC10), cyclin D1 (DCS6), myc (D84C12), AKT (9272), phospho-AKT-T308 (4056/244 F9), ERBB3 (4754) (Cell Signaling Technology); FOXA1 (Ab55-178) (Abcam); AGR2 (1C3), tubulin (B-5-1-2) (Sigma); keratin 14 (LL002, gift from Birgit Lane); ERα (Ab-16, RB-1493) (ThermoScientific); p53 (D01, gift from David Lane). After three washes in TBST, bound primary antibodies were detected by incubation with HRP-conjugated anti-rabbit, anti-mouse or anti-goat IgG (GE Healthcare) at room temperature for 1 hour, washed again in TBST, and visualized using ECL reagents (GE Healthcare). Images were captured on a Fusion FX7 scanner (Vilber Lourmat) or Hyperfilm (GE Healthcare).

### Flow cytometry analysis

Cells were grown in pWIT or svWIT medium as indicated in the figures, detached using 0.5% Trypsin-EDTA (Invitrogen), pelleted, washed and counted. 10^5^ cells were incubated for 20 minutes with the following antibodies: allophycocyanin (APC)-conjugated anti-EpCAM; phycoerythrin (PE)-conjugated anti-CD49f; PE-conjugated anti-CD10 (BD Biosciences). Non-specific PE-IgG2a and APC-IgG1 (BD Biosciences) were used as control antibodies. The gates marking the quadrants in the figures were set to exclude cells labeled by the control antibodies. Cells were washed three times then analyzed on a FACSCalibur flow cytometer (Becton Dickinson, San Jose, CA, USA) using CellQuest Pro software.

### Immunofluorescence and immunohistochemistry (IHC)

Paraffin-embedded mammary tissues were sectioned at 4 μm, deparaffinized, and boiled in pH 6.0 citrate buffer to retrieve antigens. For immunofluorescence, sections were blocked in Dako REAL Peroxidase-Blocking Solution S2023 for 10 min and incubated with primary antibodies specific for p63 (4A4; Dako, 1/40 dilution) and GFP (ab290; Abcam, 1/800 dilution) for 30 minutes. Donkey anti-rabbit IgG conjugated to Alexa Fluor AF488 (Invitrogen, 1/400 dilution) was used to detect the anti-GFP antibody. An MOM ImmPRESS Kit (MP-2400, Vector Labs) and Cy3-conjugated goat anti-horseradish peroxidase (123-165-021; Jackson ImmunoResearch Labs, 1/200 dilution) were used according to the manufacturer’s instructions to detect the anti-p63 antibody. Sections omitting the primary antibody was used as negative controls. Nuclei were visualized with DAPI, and sections were mounted with Fluoromount (Sigma). Slides were scanned on a Leica spinning disk DM6000B-CS confocal microscope. Immunohistochemical staining was performed on a Benchmark-ULTRA (Ventana) according to the manufacturer’s protocol with the following antibodies: ERα (SP1, Ventana, 32 min, pre-diluted), progesterone receptor (1E2, Ventana, 12 min, pre-diluted), p53 (DO7, Dako, 32 min, 1:50), keratin 5/6 (D5/16B4, Dako, 32 min, 1:50), keratin 14 (LL002, Ventana, 40 min + amplification, pre-diluted), keratin 8/18 (5D3, Novocastra, 20 min, 1:200), keratin 19 (A53-b/A2.26, Ventana, 32 min, pre-diluted), keratin 7 (SP52, Ventana, 32 min, pre-diluted), SMA (1A4, Sigma, 32 min, 1:12000), p63 (4A4, Dako, 32 min, 1:100), Ki67 (30-9, Ventana, 32 min, pre-diluted), GFP (B-2, Santa Cruz sc-9996, 32 min, 1:400).

### Bioinformatics

**Figure 1:**
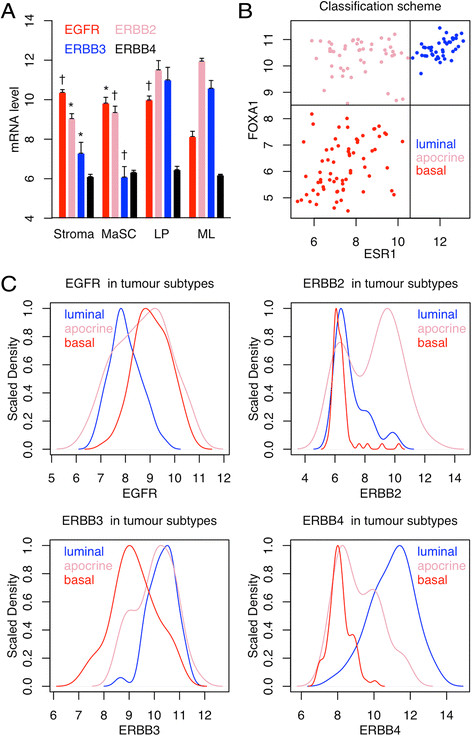
**EGFR family gene expression in normal and tumor cells. A.** Illumina gene expression data from normal human reduction mammoplasty cells (mean + SEM). MaSC, mammary stem cell; LP, luminal progenitor; ML, mature luminal. Error bars sem; † p < 0.01; * p < 0.05; n = 3; the p values are for comparisons of ML with the other groups. **B&C.** Affymetrix gene expression data for large operable or locally advanced breast cancer. The scatterplot in B shows how the tumors were classified into luminal (ER+/FOXA1+, 43 samples); molecular apocrine (ER-/FOXA1+, 49 samples); and basal-like (ER-/FOXA1-, 69 samples). The plots in **C** show the expression profile of *EGFR* family members in each tumor class. The distributions are strikingly bimodal (see text for details). The maximum density for each distribution is scaled to 1 to facilitate comparison of the different tumor classes.

To identify the normal cell types expressing *EGFR* family members in human reduction mammoplasty tissue, the most variable probes for each *EGFR* family member were selected from the Lim *et al* GSE16997 series matrix file [14]. The error bars in Figure 1A are sem; the p values shown are for t-tests comparing the mature luminal with the indicated populations. The tumor analysis in Figure 1B&C was performed on GSE6861 Affymetrix X3P gene expression data from large operable or locally advanced breast cancer [15],[16]. The CEL files were normalized with RMA in R [17], filtered to remove probesets spanning a region <56 bp, and the most variable probesets for *EGFR* family members were selected based on the standard deviation across all the tumors. The classification in Figure 1B&C into luminal, molecular apocrine and basal-like tumors is based on expression of *FOXA1* (positive in luminal and molecular apocrine tumors) and *ESR1* (positive in luminal tumors). Both genes show strongly bimodal expression; the cut-off is the nadir between the two peaks of the bimodal distributions. This classification splits breast tumors into the three groups described by Farmer *et al* [18]; *FOXA1* was used instead of *AR* because the X3P probes work slightly better (both genes are excellent markers for luminal and molecular apocrine tumors). The plot in Figure 1B shows the raw expression values generated by RMA. These values were converted to a distribution with the density command in R using the default parameters, then the maximum of each distribution was scaled to 1 to make it easier to compare the distributions in Figure 1C.

### Cell biology

**Figure 2:**
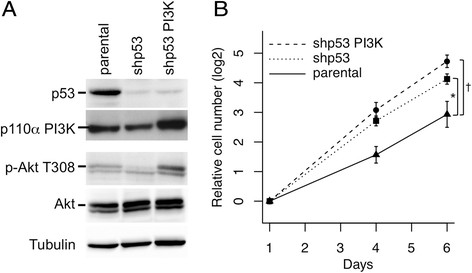
**Silencing of p53 and activation of PI3K in 4G cells. A.** Western blots showing that the shp53 vector silences p53 expression, and the *PIK3CA* vector increases PI3K expression and AKT phosphorylation on T308. **B.** Growth curves comparing parental 4G cells with cells infected with the shp53 and *PIK3CA* vectors. Error bars sem; † p < 0.001; * p < 0.05; n = 5 biological replicates.

**Figure 3:**
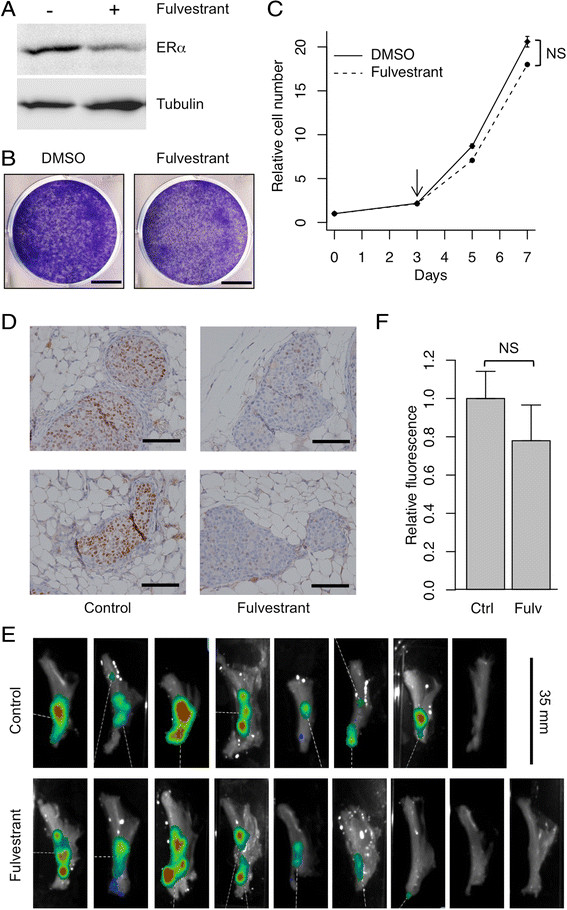
**Tumor growth is not inhibited by fulvestrant. A.** Western blot showing that treatment of 4G-shp53-PI3K tumor cells with 1 μM fulvestrant reduces the level of ERα. **B&C.** Fulvestrant has no effect on proliferation of the cells measured by crystal violet staining **(B)** or growth curves (**C**; fulvestrant treatment was started at the arrow; n = 3; NS, not significant). **D.** 4G-shp53-PI3K cells were injected into the ducts, allowed to form tumors for 3 weeks then treated with fulvestrant or vehicle for 5 weeks. Representative immunohistochemical images show a marked reduction in ERα level in treated tumors. Each image is derived from a separate animal (n = 6 for control, n = 7 for fulvestrant). The slides were all processed together. Scale bars in F, 50 μm. **E&F.** 4G-shp53-PI3K cells were injected into the ducts, allowed to form tumors for 3 weeks then treated with fulvestrant or vehicle for 3 weeks. **E.** PhotonIMAGER scans of excised mammary glands after treatment with fulvestrant or vehicle (control) to show how the response was quantified. The scanner counts fluorescence emitted by tdTomato. **F.** Quantitative data from the glands in E (n = 8 for control, n = 9 for fulvestrant; NS, not significant).

Reduction mammoplasty samples were donated by healthy premenopausal women between the ages of 21 and 40 with no previous history of breast cancer. The study was approved by the Tayside Tissue Bank ethics committee (project TR000015), and patients gave written informed consent. Breast primary epithelial cells (BPEC) were prepared by standard techniques [19] and cultured in a 37°C, 5% CO_2_, 5% O_2_ incubator on Primaria dishes (BD Biosciences) in WIT medium containing 10 ng/ml bFGF and either 10 ng/ml EGF for pWIT, or 5 ng/ml NRG1 and 10 ng/ml AREG for svWIT [2]. Low passage cultures of BPECs were sequentially infected with lentiviruses and selected for 4 to 10 days in media supplemented with 2 μg/ml puromycin, 50 μg/ml hygromycin B or 500 μg/ml G418, as appropriate. Viral particles were produced by calcium phosphate transfection of 293 T cells [20]. Infections were performed at a multiplicity of 10 infectious units per cell based on the MCF7 titer. The final 4G-shp53-PI3K cells were uniformly positive for CFP and GFP, which are carried by the BMI1 and shp53 vectors, respectively, but showed heterogeneous staining for tdTomato because the tdTomato vector was added last, not selected with an antibiotic, and confers no growth advantage on the cells. For proliferation assays, cells were treated with 2 nM 17-β-estradiol or 1 μM fulvestrant and counted at each time point with a Coulter counter. To test for significant differences between the growth curves in Figure 2B and Figure 3C, the slopes were compared after linear regression on logged data with lm in R, with a correction in Figure 2B for performing three tests.

### Xenografts

**Figure 4:**
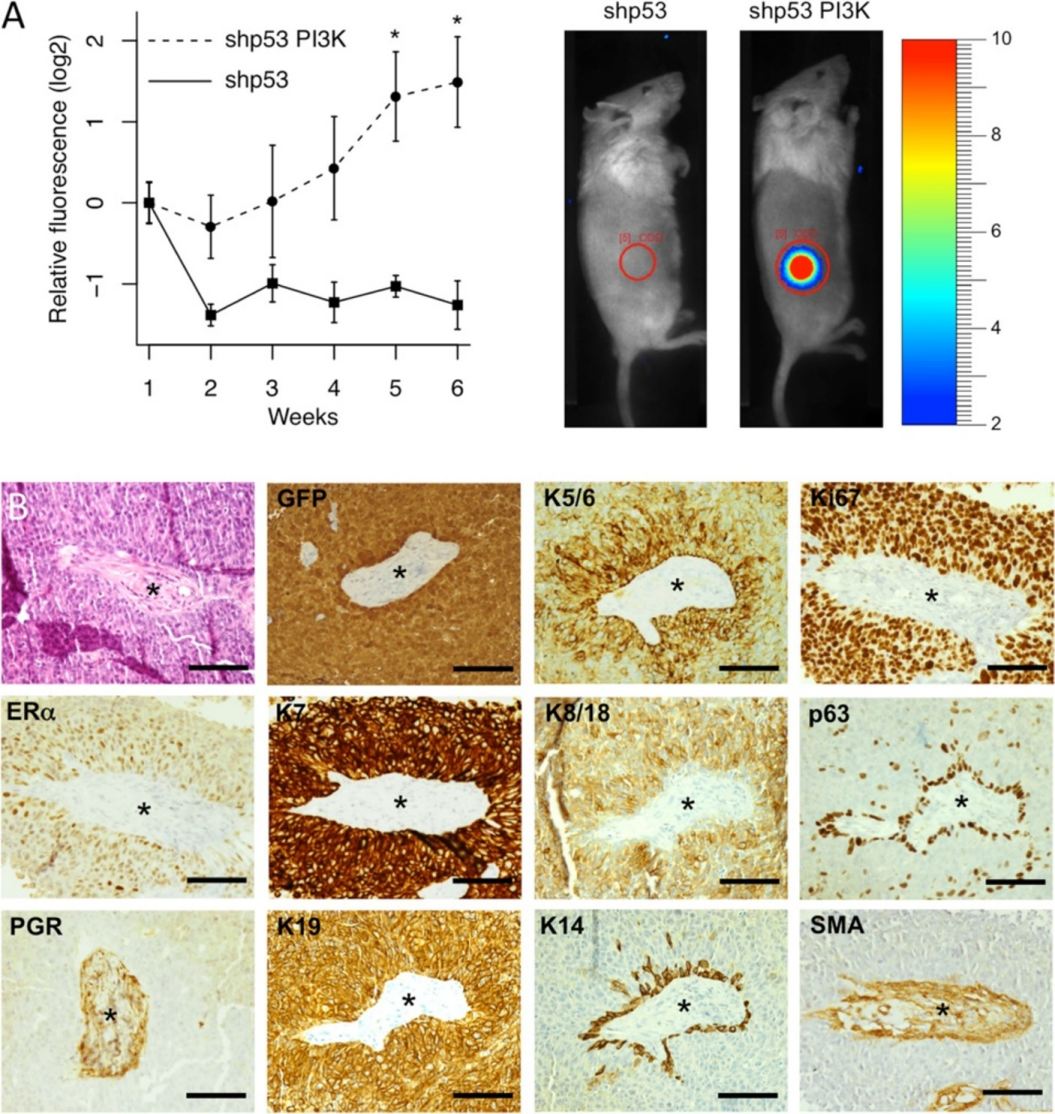
**Subcutaneous tumor formation requires activation of PI3K. A.** Red fluorescence was used to measure subcutaneous tumor growth. Fluorescence in photons per second per cm^2^ per steradian was normalized to the starting value one week after injection. n = 6; *, p < 0.05. **B.** Histopathology (H&E stain) and immunohistochemistry show that the tumors were ERα+, Ki67+ adenocarcinomas. The central region in the sections contains an island of mouse stroma surrounded by human tumor cells (PGR gives a non-specific cytoplasmic stain in the stroma). Scale bars 100 μm.

The study was performed in accordance with European Community Standards of Care and Use of Laboratory Animals under level 2 containment at the University of Bordeaux. Approval was granted for the animal experiments by the Comite d’Ethique pour l’Experimentation Animal (CEEA50) ethics committee, Bordeaux (project number 5012034-A). For xenografts, 100000 BPECs and 100000 p53-/- mouse embryo fibroblasts irradiated with 30Gy were mixed with 10% growth factor reduced Matrigel (BD Biosciences) and injected into 6-week-old female NSG mice (NOD-scid IL2RG^-/-^, Jackson Laboratory strain number 005557). The injection volume was 100 μl for subcutaneous and 10 μl for intraductal injections [10]. Tumor size after subcutaneous injection was measured with a PhotonIMAGER (Biospace, Paris, France) and scaled to the size at the first time point in each tumor group. To identify significant differences, t tests were performed in R on logged data at each time point with a correction for performing five tests in Figure 4A and Figure 3 tests in Additional file 1: Figure S1E&F. To measure tumor size after intraductal injection the glands were removed at the end of the experiment and scanned as a single group with the PhotonIMAGER.

## Results

### A new genetically defined human adenocarcinoma model

**Figure 5:**
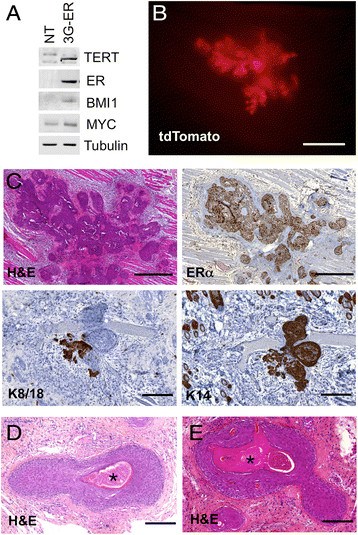
**Transformed BPECs grown in pWIT medium form squamous tumors.** Human mammary epithelial cells were infected with vectors expressing *TERT*, *BMI1*, *MYC* and *ESR1* (3G-ER cells). **A.** Western blot showing expression of the transgenes in parental (NT) and transformed cells. B&C. Tumor formation two weeks after subcutaneous injection of 3G-ER cells into NSG mice (n = 2). **B.** Stereomicrograph showing tdTomato fluorescence from the tumor. **C.** Glandular regions express keratin 8/18; squamous regions express keratin 14. All of the cells are ERα + because one of the lentiviruses used to transform the cells expresses *ESR1*. **D&E.** At 3 weeks (D, n = 2) and 5 weeks (E, n = 5) after injection, the tumors are dominated by stratified cells undergoing terminal squamous differentiation leading to the formation of keratin pearls. Keratin pearls are marked by asterisks. Scale bars: B 1 mm; upper panels in C 500 μm; lower panels in C 200 μm; D&E 200 μm.

**Figure 6:**
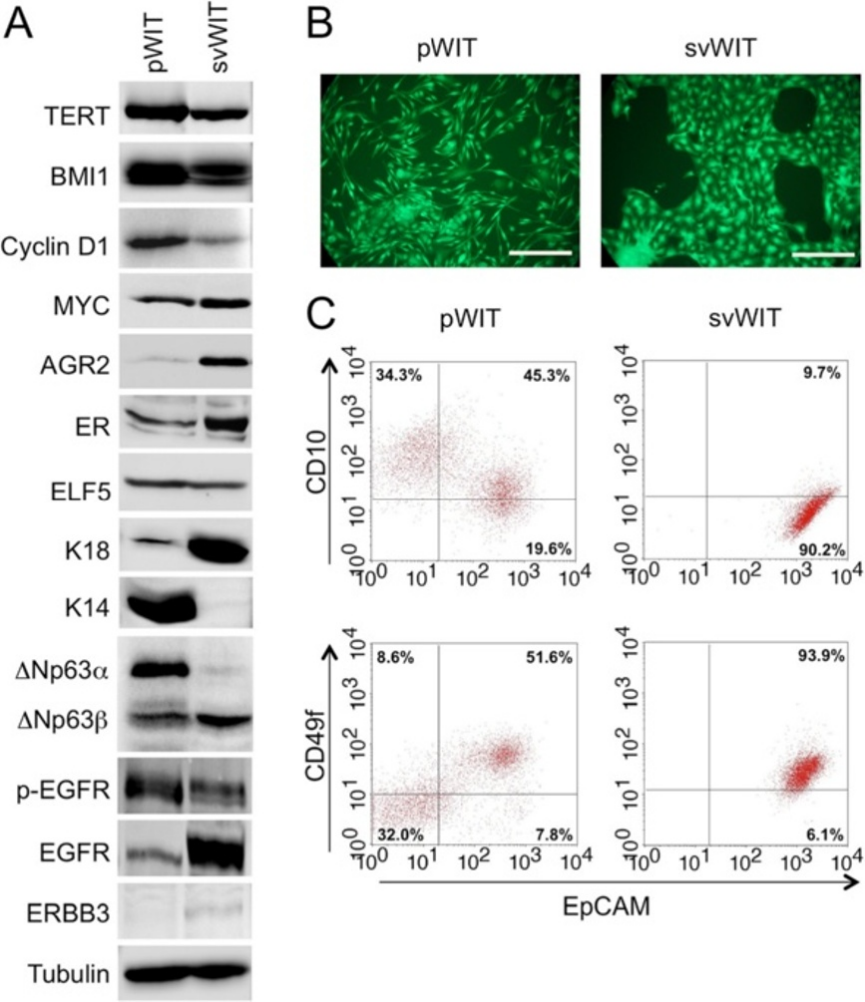
**Comparison of WIT medium containing EGF or NRG1/AREG.** Human mammary epithelial cells were infected with vectors expressing *TERT*, *BMI1*, *CCND1* and *MYC.* **A.** Western blots showing that the transgenes are expressed. The cells in medium containing AREG and NRG1 (svWIT) have luminal characteristics. The cells in medium containing EGF (pWIT) have stronger phosphorylation of EGFR. **B.** Photomicrographs showing that cells in pWIT are a mixture of spindle shaped and rounder cells, whereas cells in svWIT have a more uniform cobblestone appearance. Scale bars 250 μm. **C.** Flow cytometry shows that cells in svWIT have a putative luminal progenitor phenotype (CD49+/EPCAM+) whereas cells in pWIT are a mixture of putative luminal progenitors and myoepithelial cells. The quadrants were defined by the isotype controls.

WIT medium was developed to prevent squamous metaplasia of genetically defined mammary tumors [2]. When we grew reduction mammoplasty cells in WIT medium, transformed them with *TERT*, *BMI1*, *ESR1* and *MYC*, and injected them subcutaneously into immunodeficient mice they formed mixed tumors that rapidly became entirely squamous with the appearance of stratification and keratin pearls (Figure 5A shows expression of the transgenes; Figure 5B-E show the morphology of the tumors). *EGFR* family members show differences in expression in normal mammary epithelial cell subsets: *EGFR* expression is significantly lower in mature luminal cells than in other subsets; *ERBB3* expression is significantly lower in stem cells than in mature luminal cells; luminal progenitors have an intermediate phenotype (Figure 1A) [21]. *EGFR* family members show strikingly bimodal patterns of expression in human breast tumors: *EGFR* is higher in basal-like and molecular apocrine tumors; *ERBB2* is higher in molecular apocrine tumors; *ERBB3* is higher in luminal and molecular apocrine tumors; and *ERBB4* is higher in luminal tumors (Figure 1B&C) [15],[16],[18]. Based on these observations; on histopathological data showing *EGFR* expression by myoepithelial cells, keratinocytes and squamous tumors [22],[23]; and on the fact that EGF promotes expansion of the myoepithelial layer in short term organoid cultures [3], we reasoned that it might be possible to reduce squamous metaplasia in engineered tumor models by decreasing the activation of *EGFR* and increasing that of *ERBB3* and *ERBB4*. To test this, we grew normal reduction mammoplasty cells in WIT medium containing either EGF (“pWIT medium”) or amphiregulin (AREG) plus neuregulin (NRG1) (“svWIT medium”). Parallel cultures in the two media were infected first with *TERT* and *BMI1* viruses, then superinfected with *CCND1* and *MYC* viruses, to give “4G” cells. Expression of the transgenes was confirmed by Western blotting (Figure 6A and Additional file 2: Figure S2A). Cells grown in pWIT formed loose colonies of spindle-shaped cells mixed with larger cells, whereas cells in svWIT formed tighter colonies of more uniform round cells (Figure 6B). Flow cytometry showed that the svWIT culture was homogeneous, with a single population high in EPCAM, low in CD10 and intermediate in CD49f expression (Figure 6C). The cells in pWIT contained two populations, one CD49f-/CD10+/EPCAM-, the other CD49f+/CD10-/EPCAM+. The CD10+ population in pWIT could correspond to myoepithelial cells. The EPCAM+ cells in both media are presumably luminal cells; the EPCAM level is higher in svWIT than pWIT, suggesting that the cells are more differentiated in svWIT. Western blotting showed that the switch from EGF to AREG and NRG1 in svWIT decreased the level of EGFR receptor tail phosphorylation despite an increase in the amount of the protein (Figure 6A), consistent with weaker activation of EGFR and decreased receptor turnover. Western blotting of the cells in pWIT showed that they express keratin 14 and ΔN-p63α strongly, confirming the suggestion from the flow cytometry data that at least one of the populations in pWIT is myoepithelial or squamous. The cells in svWIT expressed luminal markers (AGR2, ERBB3 and keratin 18; Figure 6A), as well as ERα and ELF5 (Figure 6A), but not FOXA1 or GATA3 (Additional file 2: Figure S2B). ER, FOXA1 and GATA3 are markers for mature hormone-sensing cells, whereas ELF5 is a marker for the secretory lineage [24]. Coexpression of ER and ELF5, as seen in the cells grown in svWIT medium, has been proposed to mark luminal progenitors that have not yet committed to one lineage [24]. We conclude that a shift in the balance of EGFR family signaling in favor of ERBB3 selects for cells that share some features with luminal progenitors, and prevents overgrowth by myoepithelial and squamous cells.

### Tumor formation by cells grown in svWIT medium

**Figure 7:**
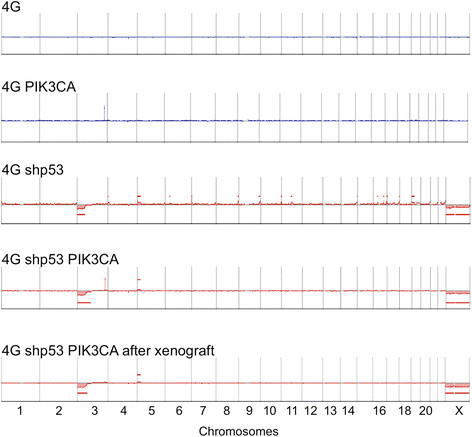
**Genomic profiles at different stages in the transformation protocol.** CGH plots of 4G cells and 4G cells infected with the *PIK3CA* vector show no copy number changes. Silencing of p53 in 4G cells leads to loss of chr X and minor changes on chromosomes 3 and 5 which do not progress after introduction of the *PIK3CA* vector. The spike on chromosome 3q in 4G PIK3CA and 4G shp53 PIK3CA cells is caused by four exonic probes in the *PIK3CA* locus. There were no additional changes after passage in mice.

It was not possible to compare the phenotype of tumors formed by 4G cells grown in the two media because the cells are not tumorigenic. To transform them fully, they were infected with lentiviruses expressing *PIK3CA-H1047R* and a small hairpin RNA (shp53) targeting *TP53*. Western blotting showed that the shp53 vector reduced the amount of p53 (Figure 2A). Cells infected with the *PIK3CA* vector showed increased expression of PI3K and increased phosphorylation of AKT on T308, indicating that PDK1 was activated by PI3K (Figure 2A). Growth curves showed that the shp53 and *PIK3CA* vectors significantly increased the growth rate (Figure 2B). As expected for genetically defined models of this type, comparative genomic hybridization (CGH) profiles were completely flat until p53 was silenced, but even cells containing all six vectors had only minor changes (loss of 3p21, gain of 5p14; loss of X; Figure 7). Interestingly, the breakpoint on 3p21 truncates *PBRM1* (*BAF180*), a known breast cancer tumor suppressor gene [25]. To determine whether the cells were tumorigenic *in vivo*, they were injected subcutaneously into immunodeficient mice. Only the cells transduced with both the shp53 and the *PIK3CA* vectors could form progressively growing tumors (Figure 4A; the images of mice show how the data were collected, the graph shows fluorescence normalized to the value one week after injection). Histological examination showed that the tumors were grade 3 adenocarcinomas (Figure 4B). The tumor cells stained strongly with anti-GFP antibody and with human-specific antibodies against keratins 7 and 19, confirming that they were carcinoma cells [26] derived from the injected human epithelial cells. Cells in contact with the mouse stroma expressed p63 and keratin 14, suggesting that they might be trying to form a myoepithelial boundary layer (Figure 4B, the stroma is marked by an asterisk). Cells in the main tumor mass expressed keratins 5/6 and 8/18. The overall pattern of keratin staining indicates that the tumors were adenocarcinomas rather than squamous carcinomas, but the presence of scattered p63-positive cells and the induction of myoepithelial genes at boundaries with mouse stroma suggest that the cells may retain some common progenitor features. Importantly, the tumor cells were positive for ERα and Ki67 but negative for PGR (Figure 4B). The experiment was repeated with two other mammoplasties (XS03 and XS05): expression of *TERT*, *BMI1*, *MYC* and *CCND1* and activation of PI3K were confirmed by Western blotting (Additional file 1: Figure S1A&B); flow cytometry showed the presence of a single EPCAM+ luminal population (Additional file 1: Figure S1C&D); and subcutaneous xenografts only formed tumors following introduction of the *PIK3CA-H1047R* vector (Additional file 1: Figure S1E&F). We conclude that cells transformed in medium containing AREG and NRG1 form luminal B mammary adenocarcinomas with squamous metaplasia of cells in contact with the mouse stroma.

### Orthotopic tumor formation

Behbod and colleagues recently described a new approach to model DCIS by injecting cells through the nipple directly into the mouse mammary ductal tree [10]. To test whether exposure to this more physiological context would correct the transformed phenotype of the 4G-shp53-PIK3CA cells we mixed them with irradiated mouse fibroblasts and laminin-rich extracellular matrix (matrigel) and injected the mixture into the ducts. Fibroblasts and matrigel were used to promote the survival of the epithelial cells in the perioperative period, but we have not formally tested whether they increase the rate of engraftment; in contrast with the Kuperwasser approach the fibroblasts were murine in origin and injected into the ducts not the stroma. Six week-old mice were injected because the majority of the primary ductal tree is present, providing a large surface area for the human cells to colonize, but the ducts are still growing and forming side branches at this age, so the cells can potentially be incorporated into ducts as they are laid down.

**Figure 8:**
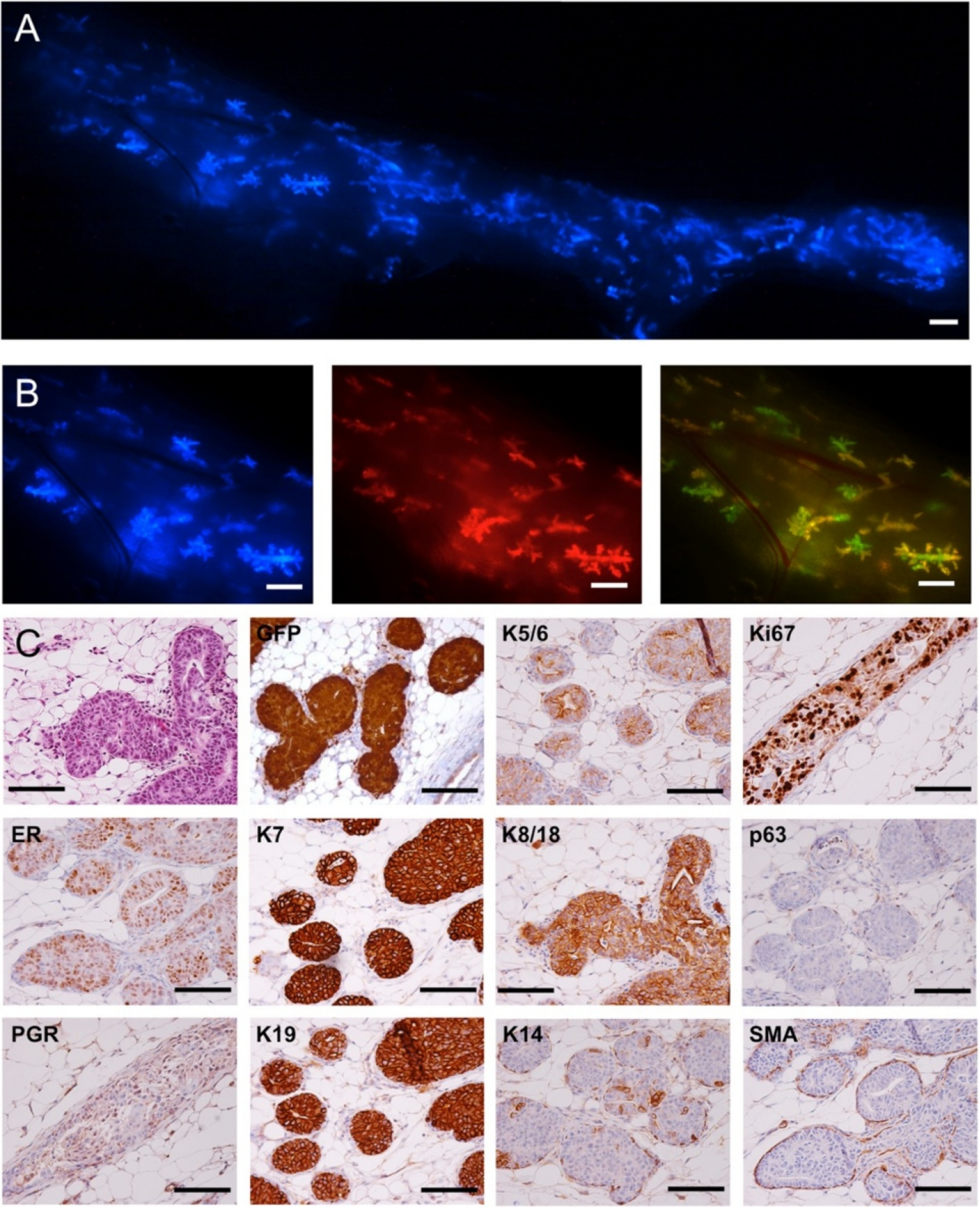
**DCIS formation 3 weeks after intraductal injection of 4G-shp53-PI3K tumor cells. A.** Composite image showing human cells expressing CFP scattered throughout the gland. **B.** Enlarged images; the right panel shows alternating clusters of yellow and green cells. **C.** H&E stain and immunohistochemistry showing ERα+, Ki67+ DCIS (the PGR staining is negative in the tumor cell nuclei relative to controls on the slide). Scale bars A & B 1 mm, C 100 μm.

**Figure 9:**
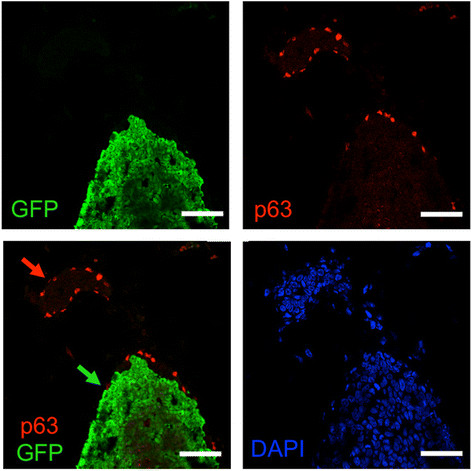
**The myoepithelial layer in DCIS is murine in origin.** Ducts containing DCIS 3 weeks after intraductal injection of 4G-shp53-PI3K tumor cells were costained for p63 (red), GFP (green) and DNA (DAPI, blue) (n = 4). The p63-positive cells do not stain for GFP, indicating that they are murine in origin. Red arrow, normal murine duct; green arrow, DCIS. Scale bars 50 μm.

Three weeks after injection, mice were sacrificed and the mammary glands were examined by stereomicroscopy (Figure 8A). Clusters of fluorescent cells were scattered throughout the injected glands. At higher magnification, the main ducts and side branches are clearly visible (Figure 8B). The injected cells expressed red, blue and green fluorescent proteins but the strength of the red signal was variable in different clones because the number of copies of the tdTomato provirus varied between cells. Clusters of yellow cells alternate with clusters of green cells in the right panel in Figure 8B, indicating that different ratios of red and green vector were present; we can infer that hundreds of cells successfully seeded independent tumor foci throughout the gland. The H&E stain showed that the mammary ducts were filled with cells resembling the pattern seen in DCIS (Figure 8C). IHC for GFP confirmed that the tumor cells were derived from the injected human cells. IHC for p63 and SMA showed that at this stage (3 weeks after injection) the myoepithelial layer was present (Figure 8C), indicating that the tumor was DCIS, but there were fewer myoepithelial cells than in normal murine ducts (Figure 9). As expected, the myoepithelial cells did not stain for GFP, indicating that they were murine in origin (Figure 9: red arrow, normal duct; green arrow, DCIS). Intraductal injection of cells from the other mammoplasties also produced ERα + DCIS (Additional file 1: Figure S1G). The tumor cells were all positive for keratins 7, 8/18 and 19, most were weakly positive for keratins 5/6, and occasional cells were positive for keratin 14 (Figure 8C). This mixed luminal/basal pattern has been reported to occur in one quarter of primary operable breast tumors [27]. Interestingly, the cells were positive for ERα and Ki67 but negative for PGR (Figure 8C; the PGR stain is negative relative to controls cells on the same section).

**Figure 10:**
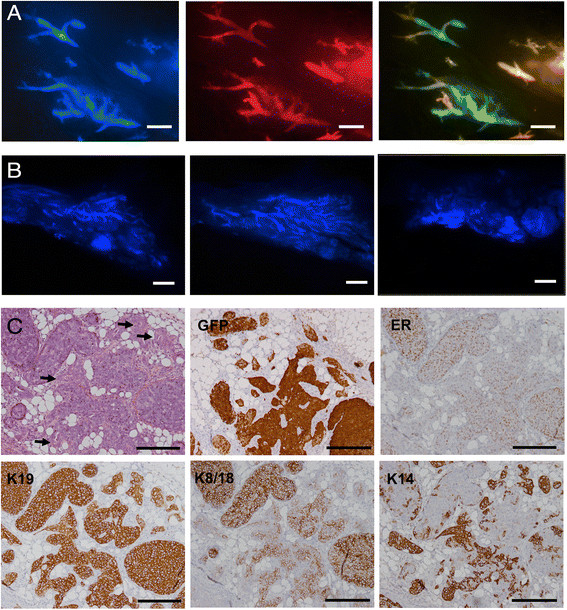
**Invasive tumor formation 6 and 8 weeks after intraductal injection of 4G-shp53-PI3K tumor cells. A.** Mammary ducts dilated by human tumor cells at 6 weeks. **B.** Diffuse blue masses 8 weeks after intraductal injection caused by tumor cells invading the stroma. **C.** Mixed DCIS and invasive tumor at 8 weeks. H&E stain and immunohistochemistry show increased keratin 14 staining as cells migrate from the ducts into the stroma. Early squamous changes are marked by arrows. There are small difference in the structures in the serial sections in C because the structures gradually change as the sectioning proceeds through the paraffin block. Scale bars A 1 mm, B 2 mm, C 100 μm.

Six weeks after injection, stereomicroscopy showed dilated ducts packed with fluorescent cells. There was again variable red staining, indicating that multiple clones were present, but large regions showed homogeneous staining, suggesting that individual clones had expanded to fill those regions (Figure 10A). Eight weeks after injection, tumor had spread to fill large contiguous parts of the ductal tree (Figure 10B). H&E staining showed that the tumor had broken out of the ducts and started to invade the mammary fat (Figure 10C). Keratin 19 and GFP staining confirmed that the tumor cells were derived from the injected human cells. The tumor cells still expressed ERα but there was lower keratin 8/18 and higher keratin 14 staining as the cells migrated from the ducts into the stroma. This was accompanied by histological features of early squamous differentiation (pink cytoplasmic staining, marked by arrows in Figure 10C, without stratification or keratin pearls). We conclude that loss of environmental cues when the tumor breaks free from the ducts allows the cells to evolve towards a more adeno-squamous phenotype, although the squamous differentiation was much less pronounced than in our previous model [12].

Since the tumors are potentially a model for high grade ERα-positive breast cancer, they were tested for estrogen-dependence. Western blotting showed that *in vitro* treatment with fulvestrant reduced the level of ERα (Figure 3A), but the growth rate was unaffected (Figure 3B&C). The cells were xenografted intraductally into mice and allowed to form tumors for 3 weeks, after which the mice received weekly injections of fulvestrant for 5 weeks. Immunohistochemistry showed that the level of ERα was lower in the tumors that received fulvestrant than in the vehicle-treated controls (Figure 3D), confirming that the drug had reached its target *in vivo*. To quantitate the response, mice were allowed to form tumors for 3 weeks, treatment was given for 3 weeks, the injected mammary glands were excised, and total tdTomato fluorescence was measured (Figure 3E). There was no significant difference between the treated and control groups (Figure 3F). The lack of response *in vitro* and *in vivo* mimics the resistance of human luminal B tumors to endocrine therapy in patients with breast cancer.

### Humanization of the mouse mammary gland

**Figure 11:**
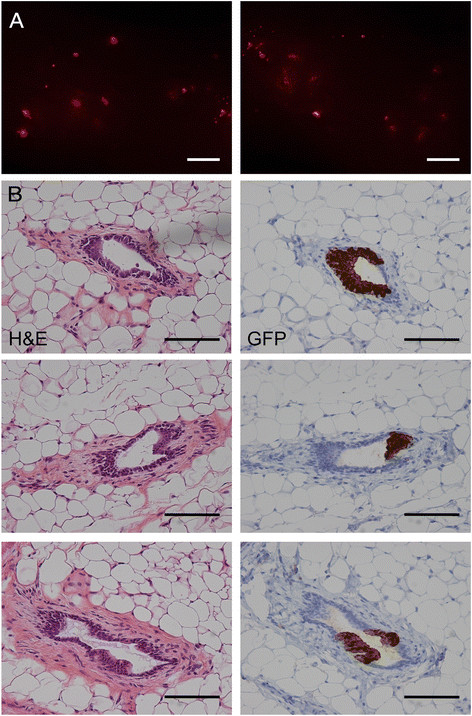
**Foci of engrafted human cells 3 weeks after intraductal injection of 4G-shp53 cells. A.** Stereomicrograph showing isolated foci of human cells (the images cover a single gland with a small overlap in the middle). **B.** Attachment of 4G-shp53 cells to the duct wall 3 weeks after intraductal injection. Left panels, H&E staining. Right panels, matched sections showing GFP staining to identify the human cells. The clumps of human cells correspond to the scattered foci of fluorescent cells seen by stereomicroscopy in A. Scale bars A 1 mm, B 100 μm. n = 2.

**Figure 12:**
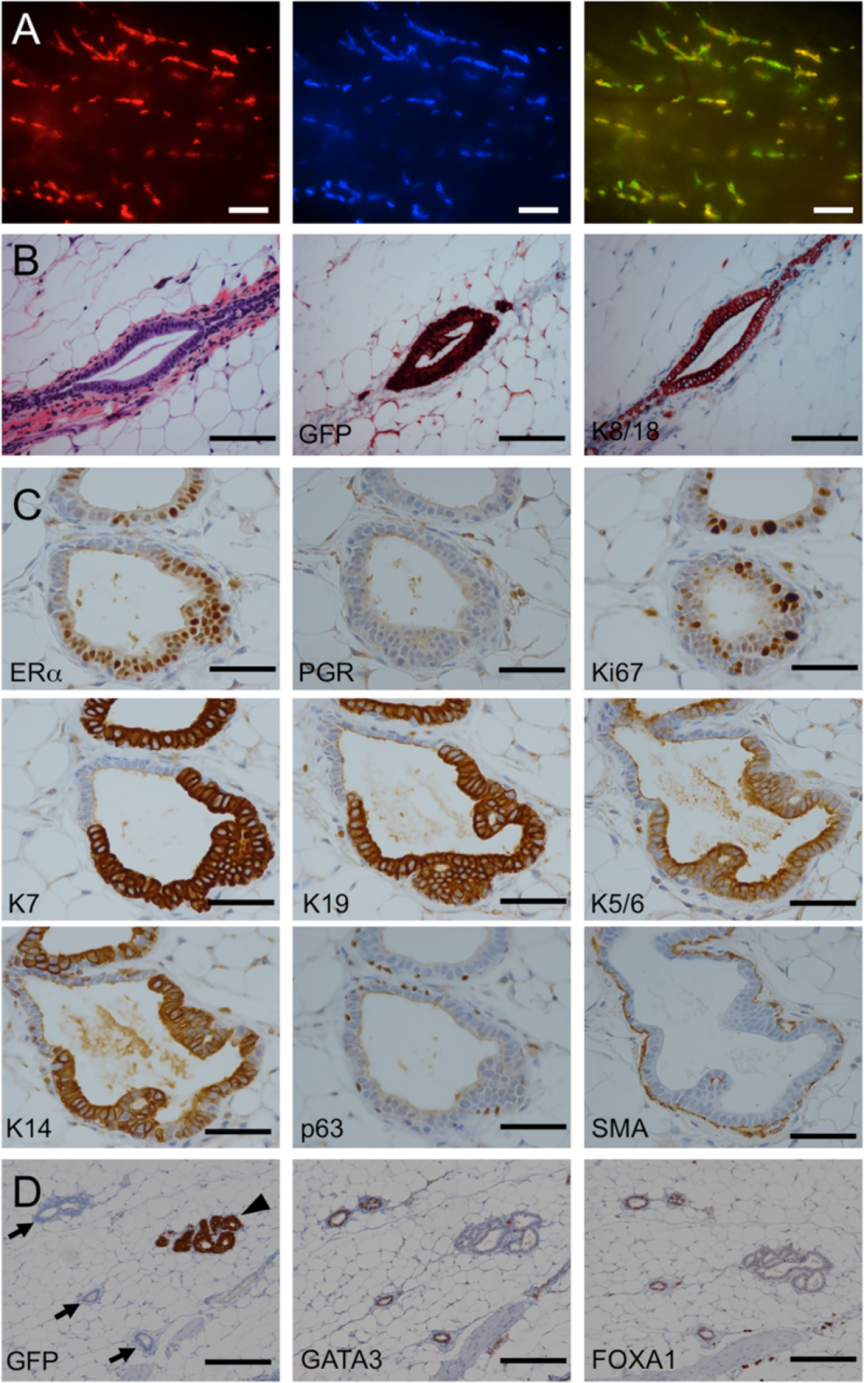
**Replacement of the murine luminal layer 6 weeks after intraductal injection of 4G-shp53 cells. A.** Stereomicrograph showing spread of human cells within the ducts. Alternating yellow and green areas in the right panel demonstrate independent engraftment of multiple clones. **B&C.** Histopathology (H&E stain) and immunohistochemistry show that the human cells have replaced the murine luminal layer with morphologically normal human cells. The human cells are larger, as seen at junctions between murine and human luminal cells. GFP, keratin 7 and keratin 19 staining specifically label the human cells. The cells are ERα+, PGR-, Ki67+ luminal cells. SMA staining shows that the myoepithelial layer is intact. **D.** GATA3 and FOXA1 are positive in murine ducts (marked by arrows) but negative in humanized ducts (marked by an arrowhead). Scale bars A 1 mm, B 100 μm, C 50 μm, D 200 μm.

**Figure 13:**
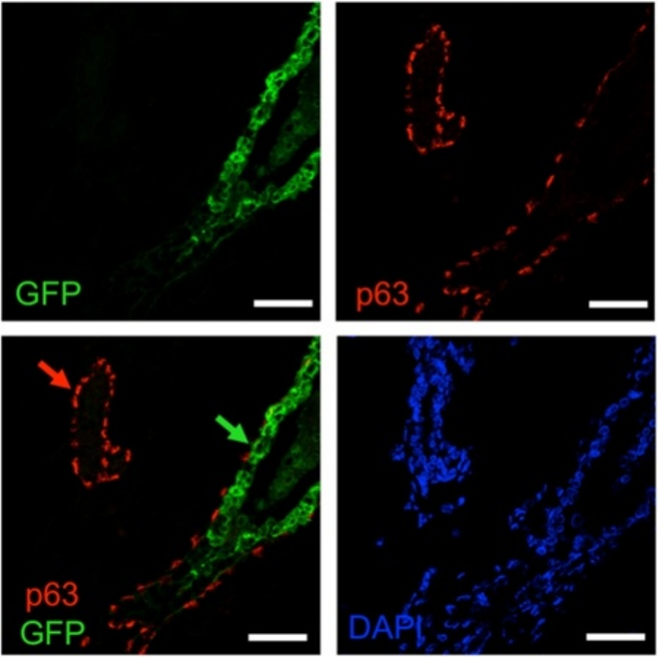
**The myoepithelial layer is murine in humanized glands.** Ducts humanized with 4G-shp53 cells were costained for p63 (red), GFP (green) and DNA (DAPI, blue) (n = 3). All injected human cells express GFP strongly. Green arrow, human luminal cells in a humanized duct; red arrow, murine myoepithelial cells in a murine duct. Scale bars 50 μm.

**Figure 14:**
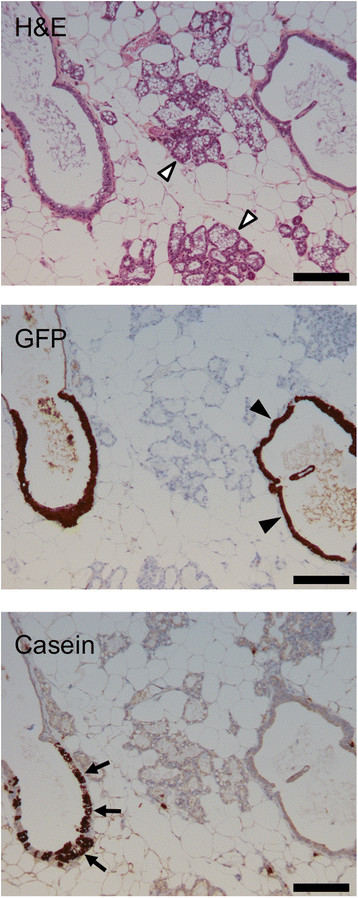
**4G-shp53 cells can undergo secretory differentiation but do not form normal alveoli.** Mice with human cells engrafted in the ducts were sacrificed on day 1 of lactation (n = 2). Histopathology (H&E stain) and immunohistochemistry for GFP show that human cells are present in the ducts but do not spread into the lactating alveoli. Immunohistochemistry for casein shows that some of the human cells in the ducts have undergone secretory differentiation. The structures in the middle of the image are mouse alveoli (open arrowheads); they do not stain for casein because the antibody is human specific. The structure on the right is a humanized duct containing cells that do not express casein (closed arrowheads). The structure on the left is a humanized duct containing cells that do express casein (arrows). Scale bars 200 μm.

The Weinberg lab has shown that “humanization” of the mouse mammary gland by human fibroblasts promotes engraftment of human mammary epithelial cells [28]. The epithelial cells form duct-like structures but they do not ramify to fill the gland with a normal ductal tree, possibly because it is difficult to distribute human fibroblasts evenly throughout the fat pad. The logic behind their approach is that subtle differences in signaling proteins create a species barrier that prevents human myoepithelial cells from communicating properly with mouse fibroblasts. We reasoned that human luminal cells might be able to communicate effectively with mouse myoepithelial cells and therefore tried to use the mouse myoepithelial layer as a scaffold upon which to build a human luminal epithelium. We initially attempted to humanize the ducts of NSG mice by intraductal injection of reduction mammoplasty cells immortalized by *TERT* and *BMI1* (“2G” cells), but the cells were rapidly lost. To give the human cells a proliferative advantage over the resident murine luminal cells, we tested 4G-shp53 cells, which we showed above to be non-tumorigenic. Three weeks after intraductal injection there was much less fluorescence in the injected glands than after injection of their *PIK3CA*-transformed counterparts (Figure 11A). Histological examination showed that small clumps of human cells had penetrated the mouse luminal layer and attached to the underlying murine myoepithelial layer (Figure 11B). By six weeks, large segments of the mammary ductal tree were fluorescent, indicating that viable human cells had engrafted and increased in number. The presence of alternating yellow and green regions showed that the grafts were derived from multiple different clones (Figure 12A). Immunohistochemistry for GFP confirmed that extensive regions of the ductal tree contained human cells (Figure 12B). Histologically, the human cells were slightly larger than the murine luminal cells but there was no evidence of the premalignant changes of the sort usually observed in flat epithelial atypia or atypical ductal hyperplasia [29]. Human cells replaced murine cells in the luminal layer but the myoepithelial layer was murine, as shown by the absence of cells positive for both p63 and GFP in double labeled sections (Figure 13). The myoepithelial layer was intact, as judged by staining for smooth muscle actin, but the number of myoepithelial cells was lower than in contiguous regions with murine luminal cells. All of the human cells expressed ERα, and many expressed Ki67, but they did not express PGR, FOXA1 or GATA3 (Figure 12C&D), consistent with their *in vitro* profile. To test whether the cells could differentiate to form secretory cells, two mice were grafted, allowed to form humanized ducts, then mated and sacrificed on day 1 of lactation, 14 weeks after intraductal injection of the human cells. The human cells were restricted to the ducts and did not form alveoli although some human cells in the ducts did undergo secretory differentiation (Figure 14: open arrowheads show murine alveoli; filled arrowheads show a non-secretory humanized duct; arrows show casein-expressing cells in a humanized duct). Taken together, the data suggest that the cells immortalized by the *TERT*, *BMI1*, *CCND1*, *MYC* and shp53 transgenes may be precursors of hormone sensing or secretory cells trapped at the luminal progenitor stage.

## Discussion

We have shown that genetically defined human mammary epithelial cells expressing multiple oncogenes can form a morphologically normal luminal layer in the mouse mammary gland, and that expression of a single additional activated oncogene, *PIK3CA-H1047R*, is sufficient to convert them into invasive ERα + adenocarcinoma cells. Two factors were probably critical to the success of the approach: the use of oncogenes commonly mutated or amplified in ERα + breast cancer, and the use of culture medium favoring ERBB3 over EGFR signaling to prevent squamous metaplasia *in vitro*.

Previous attempts to make breast cancer models by transformation of normal human mammary epithelial cells typically led to the formation of overtly squamous tumors that are unrepresentative of breast cancers seen in human patients. Several arguments implicate *EGFR* signaling in squamous metaplasia. First, *EGFR* is prominently expressed in the skin [22]. Second, the myoepithelial layer proliferates at the expense of the luminal layer in short term organoid culture in media containing commonly used levels of EGF [3]. Third, deletion of *Erbb3* in mice leads to loss of the luminal layer and expansion of the basal/myoepithelial layer [30]. Fourth, *EGFR* is preferentially expressed by stem cells and basal-like tumors, whereas *ERBB3* is expressed by mature luminal cells and luminal tumors (luminal progenitors express both receptors, presumably because of their intermediate status; Figure 1 and refs [21],[30]). To attenuate EGFR signaling we replaced EGF in the culture medium with amphiregulin (AREG), which is 100-fold less active on EGFR [3], and added neuregulin (NRG1), which activates ERBB3 and ERBB4 reviewed in ref [31]. The WIT medium we used was developed specifically to promote the growth of luminal cells [2], but in our conditions, cell lines established in EGF-containing WIT medium underwent squamous metaplasia. Furthermore, our cell lines established in AREG/NRG1 medium grow poorly if transferred back into EGF medium, suggesting that the AREG/NRG1 combination not only prevents overgrowth of a confused myoepithelial population but also favors the growth of luminal cells. That said, we fully agree with Ince *et al* [2] that WIT medium favors the growth of luminal cells; we suspect that the differences between our results and theirs reflect subtle differences in the formulation of the medium, the origin of the cells or the choice of transforming genes. Whatever the reason, our results indicate that replacement of EGF with AREG and NRG1 is a simple modification that makes WIT into an even more robust luminalizing medium.

Transgenic mouse models frequently produce tumors with histological features rarely seen in human breast cancer patients. The striking feature of PI3K-induced mouse models is the extent to which they express ERα, despite their unpromising histology [32]. This points to a role for PI3K in the regulation of ERα expression, but the cells in our model already formed ERα + xenografts before infection with the *PIK3CA* vector. The Kuperwasser group has described a model based on *CCND1*, *PI3K, KRAS* and *TP53* that produces ERα + tumors [4],[5]. *CCND1* is a strong candidate for driving ERα + tumor formation in both our models, since it is commonly amplified in ERα + tumors and activates ERα in the absence of hormone [33],[34]. In addition to the above arguments, the rationale for using *CCND1*, *PI3K-H1047R*, *MYC*, *TERT*, *BMI1* and *sh-p53* was based on genomic data in luminal breast cancer and on past experience with mammary transformation protocols. According to the TCGA consortium, *PIK3CA* mutations are seen in 45% of luminal A and 29% of luminal B tumors; *TP53* mutations are seen in 29% of luminal B tumors [35]. *MYC* was included in the transformation cocktail because Elenbaas *et al* reported that the endogenous *MYC* gene would amplify spontaneously if *MYC* was not supplied exogenously in their transformation protocol [36]; and *TERT* was included to prevent telomere erosion [1]. *BMI1* is preferentially expressed by luminal tumors [12],[37], and cooperates well with *MYC* [38], but it was included mainly to prevent unwanted differentiation or genomic instability at early times after the cells were put into culture [12],[39]; it is possible that in the new medium it may not be necessary. BMI1 prevents activation of the p53 and Rb pathways [40], but the fact that silencing of p53 by RNA interference is required for full transformation in our model indicates that BMI1 is having at best a partial effect; we suspect this is because *BMI1* is expressed at a low level from our lentiviral vector. After silencing of p53, the cells from the XS11 mammoplasty acquired a deletion on 3p21 starting within *PBRM1*, the gene encoding the BAF180 subunit of the PBAF ATP-dependent chromatin-remodeling complex [41]. Truncating mutations, as seen here, have previously been described in breast cancer, where they reduce p21 induction by TGFβ and p53 [25]. We have not performed CGH on multiple independently derived cell lines so we can not say whether this change is absolutely required for transformation with *CCND1*, *PI3K-H1047R*, *MYC*, *TERT*, *BMI1* and *sh-p53*, but it seems unlikely. Indeed, the more striking feature is how flat the profiles are after transformation with defined oncogenes, something we also saw in our previous ER+ model [12].

Despite robust ERα expression there was no response to fulvestrant. *CCND1* and mutant *PIK3CA* can confer resistance to endocrine therapy in cell lines but the relationship between *PIK3CA* mutation and the response to endocrine therapy is hotly debated [42]. Paradoxically, *PIK3CA* mutations are associated with a gene signature of low mTORC1 signaling and better outcomes in ERα + breast cancer [43]. It will be interesting to see whether the genetically defined ERα + *PIK3CA*-mutant model we have developed can shed fresh light on this problem.

Genomic studies indicate that DCIS has a similar mutation profile to invasive cancer [44], and molecular clock studies indicate that breast tumors undergo little clonal expansion or genomic rearrangement until the last driver mutation is acquired [45]. Taken together, these results push the timeline for acquisition of oncogenic mutations far before the formation of DCIS. Our data following intraductal injection of 4G-shp53 cells would say that cells with all but the last driver mutation can form a morphologically normal luminal epithelium. The 4G-shp53 cells have a growth advantage over the murine luminal cells they replace but, apart from being bigger than murine luminal cells, they are normal in appearance. They do not survive after subcutaneous injection, indicating that mammary ducts provide a more welcoming environment than skin. The microenvironment of the duct seems to promote glandular differentiation: the transformed cells were predominantly keratin 18+/keratin 14- while confined to the ducts, but keratin 14 expression increased when they migrated into the stroma. A possible interpretation is that the murine stroma is programmed to release EGF in amounts appropriate for murine progenitors but excessive for human progenitors [3]. Alternatively, the fact that occasional human cells bordering the necrotic core of the largest DCIS lesions expressed p63 suggests that squamous differentiation may be a cell autonomous effect that normal myoepithelial cells prevent through release of a diffusible factor.

We see humanization of the luminal layer in the mouse ducts after intraductal injection as a form of tissue engineering where the murine myoepithelial layer serves as a scaffold on which to build a human luminal layer. The failure of human cells to form a normal mammary ductal tree after xenografting into the mouse mammary fat pad is normally explained by the existence of a species barrier between human myoepithelial cells and mouse stromal cells. The Weinberg group has shown that this barrier can be partially overcome by infiltration of the mouse stroma with human fibroblasts [28]. Our results indicate that it may be better to retain the mouse myoepithelial layer and replace only the luminal layer with human cells. To do this we used an intraductal xenografting approach developed by Behbod and colleagues, who showed that the approach is strikingly effective for growth of primary human DCIS cells in the mouse [10]. The DCIS histology in the mouse faithfully recapitulated the histology of the human sample from which it was derived, and included regions where hyperplastic human cells formed single and multi-layered epithelia [11]. Taken together, our results suggest that intraductal grafting is a major advance over previous approaches to model human breast cancer biology in the mouse. We were not able to replace the murine luminal layer with unmodified human cells. Instead we started with fully transformed cells and removed one oncogene, PIK3CA. Although the 4G-shp53 cells were able to form a morphologically normal luminal layer in the ducts, they were clearly not functionally normal because they could not form normal alveolar structures in lactating mice. It will be interesting to test cells expressing even fewer oncogenes to determine the point at which human cells lose their competitive advantage over mouse cells, in order to see whether they can fully humanize the gland and produce functioning alveoli.

A weakness of mouse transgenic models in general is the strong tendency for tumor histology not to resemble that of human breast cancer. One reason may be a fundamental difference in the cell of origin of murine and human mammary tumors. Recent work in the Blanpain lab has identified a long-lived luminal stem cell [6] whose human homolog is a strong candidate for the cell of origin of luminal, molecular apocrine and basal-like human breast cancers. The ERα + ELF5+ cell in our cultures could be the preneoplastic counterpart of this cell. Recent work from the Ormandy lab indicates that ERα and ELF5 cross-inhibit one another in luminal progenitors, with definitive hormone-sensing or secretory lineage differentiation ensuing when one factor triumphs over the other [24]. This is perfectly consistent with the failure of our transformed cells to respond to inhibition of ERα with fulvestrant. Drawing parallels between normal lineages and tumor cells is fraught with difficulty because tumor cells inevitably fail many of the tests cell biologists use to characterize normal cells, such as differentiation of progenitors into mature, terminally differentiated cells. Nevertheless, taken together, our data suggest that injection of genetically engineered human cells into the mouse mammary ducts may provide a strategy to study the differentiation of normal and malignant human mammary epithelial cells in a more physiological context than has hitherto been possible.

## Conclusion

In conclusion, we have shown that by altering the balance of EGFR family signaling in favor of ERBB3/ERBB4 we can prevent squamous metaplasia of mammary epithelial cells *in vitro*, that human cells with multiple defined oncogenic changes can replace the luminal cell layer in the mouse mammary gland with a morphologically normal luminal layer of human cells, and that addition of a single activated oncogene, mutant PIK3CA, is sufficient to convert these cells into invasive ERα + adenocarcinoma cells.

## Additional files

## Electronic supplementary material

Additional file 1: Figure S1: **Transformation in svWIT with different mammoplasties (XS03 and XS05).** A&B. Western blots showing expression of the *TERT*, *BMI1*, *MYC* and *CCND1* transgenes (A) and phosphorylation of AKT on T308 induced by PI3K (B). C&D. Flow cytometry shows the formation of single populations of EPCAM+ cells after transformation (C, XS05; D, XS03). The quadrants were defined by the isotype controls. E&F. 4G-shp53 cells are unable to survive after subcutaneous xenografting. Only the cells superinfected with the *PIK3CA* vector survive and form tumors (E, XS05; F, XS03; n = 5). G. DCIS after intraductal injection of XS05 4G-shp53-PI3K cells (H&E staining and IHC for ERα Ki67, GFP, p63, SMA and keratins). Scale bars 100 μm. (TIFF 8310 kb) (TIFF 8 MB)

Additional file 2: Figure S2: Western blots showing expression of the transgenes in 4G and parental cells (A) and lack of FOXA1 and GATA3 expression in 4G cells (B). MCF7 cells were used as a positive control for the FOXA1 and GATA3 blots. p4-p6, different passages of the 4G cells in svWIT medium. (TIFF 1460 kb) (TIFF 1 MB)

Below are the links to the authors’ original submitted files for images.

Authors’ original file for figure 1

Authors’ original file for figure 2

Authors’ original file for figure 3

Authors’ original file for figure 4

Authors’ original file for figure 5

Authors’ original file for figure 6

Authors’ original file for figure 7

Authors’ original file for figure 8

Authors’ original file for figure 9

Authors’ original file for figure 10

Authors’ original file for figure 11

Authors’ original file for figure 12

Authors’ original file for figure 13

Authors’ original file for figure 14

## Abbreviations

4G cells: BPECs transduced with *TERT*, *BMI1*, *MYC* and *CCND1* lentiviral vectors
4G-shp53 cells: 4G cells transduced with *sh-TP53* lentiviral vector
4G-shp53-PI3K cells: 4G-shp53 cells transduced with *PIK3CA-H1047R* lentiviral vector
Addgene: Plasmid repository (http://www.addgene.org)
AKT: v-akt murine thymoma viral oncogene
AREG: Amphiregulin
BMI1: B lymphoma Mo-MLV insertion region 1 oncogene
BPEC: Breast primary epithelial cell
CCND1: Cyclin D1
CD10: membrane metallo-endopeptidase (*MME*, CALLA)
CD49f: integrin alpha 6 (*ITGA6*)
CFP: Cyan fluorescent protein
CGH: Comparative genomic hybridization
DCIS: Ductal carcinoma in situ
EGF: Epidermal growth factor
EGFR: Epidermal growth factor receptor
ELF5: E74-like factor 5 (ets domain transcription factor)
EPCAM: Epithelial cell adhesion molecule
ERBB2: EGFR homolog 2 (v-erb-b2 avian erythroblastic leukemia viral oncogene homolog 2)
ERBB3: EGFR homolog 3 (v-erb-b2 avian erythroblastic leukemia viral oncogene homolog 3)
ERBB4: EGFR homolog 4 (v-erb-b2 avian erythroblastic leukemia viral oncogene homolog 4)
ESR1/ERα: Estrogen receptor alpha
FOXA1: Forkhead box A1 transcription factor
GATA3: GATA binding protein 3 transcription factor
GFP: Green fluorescent protein
IHC: Immunohistochemistry
Ki67: Antigen encoded by the *MKI67* gene, recognized by the Ki67 antibody
mTORC1: mammalian target of rapamycin complex 1
NRG1: Neuregulin 1
NSG: Non-obese diabetic (NOD), severe combined immunodeficiency (SCID), interleukin 2 receptor gamma chain-null (IL2RG-/-) mouse
PBAF: Polybromo and BRG1-associated factor chromatin remodeling complex
PDK1: Pyruvate dehydrogenase kinase isoenzyme 1
PGR: Progesterone receptor
PIK3CA: Phosphatidylinositol-4,5-bisphosphate 3-kinase catalytic subunit alpha
PlasmID: Plasmid repository (http://plasmid.med.harvard.edu)
pWIT: WIT medium for primary cells, contains 10 ng/ml EGF
R: Open source statistical software (http://cran.r-project.org)
RMA: Robust multi-array average gene expression measure
SDS-PAGE: sodium dodecyl sulfate polyacrylamide gel electrophoresis
SMA: Smooth muscle actin
svWIT: WIT medium containing 5 ng/ml NRG1 and 10 ng/ml AREG
tdTomato: Red fluorescent protein
TERT: Telomerase catalytic subunit
TGFβ; TP53: Tumor protein p53
WIT: Mammary culture medium developed by Tan Ince (ref [2])
