# Erratum to: Humanization of the mouse mammary gland by replacement of the luminal layer with genetically engineered preneoplastic human cells

**DOI:** 10.1186/s13058-015-0612-1

**Published:** 2015-09-16

**Authors:** Stephanie Verbeke, Elodie Richard, Elodie Monceau, Xenia Schmidt, Benoit Rousseau, Valerie Velasco, David Bernard, Herve Bonnefoi, Gaetan MacGrogan, Richard D Iggo

**Affiliations:** INSERM U916, Bergonié Cancer Institute, University of Bordeaux, 229 cours de l’Argonne, Bordeaux, 33076 France; School of Medicine, University of St Andrews, Medical and Biological Sciences Building, North Haugh, St Andrews, KY16 9TF UK; Animalerie A2, University of Bordeaux, 146 rue Léo Saignat, Bordeaux, 33076 France; Pathology Department, Bergonié Cancer Institute, 229 cours de l’Argonne, Bordeaux, 33076 France; INSERM U1052, Centre Leon Berard, University of Lyon, 28 rue Laennec, Lyon, 69008 France

## Erratum

Due to a typesetting error, the labelling was changed and the figures in this article [1] were presented in the order 2, 4, 10, 6, 1, 3, 5, 7, 8, 9, 11, 12, 13, 14 and the supplementary figure links were inverted. The revised version has the figures in the correct order.
