# Supplementary figures and images for: Humanization of the mouse mammary gland by replacement of the luminal layer with genetically-engineered preneoplastic human cells
Source: Breast Cancer Res. 2014 Dec 20;16:504. doi: 10.1186/s13058-014-0504-9 (PMC4407301; doi:10.1186/s13058-014-0504-9)

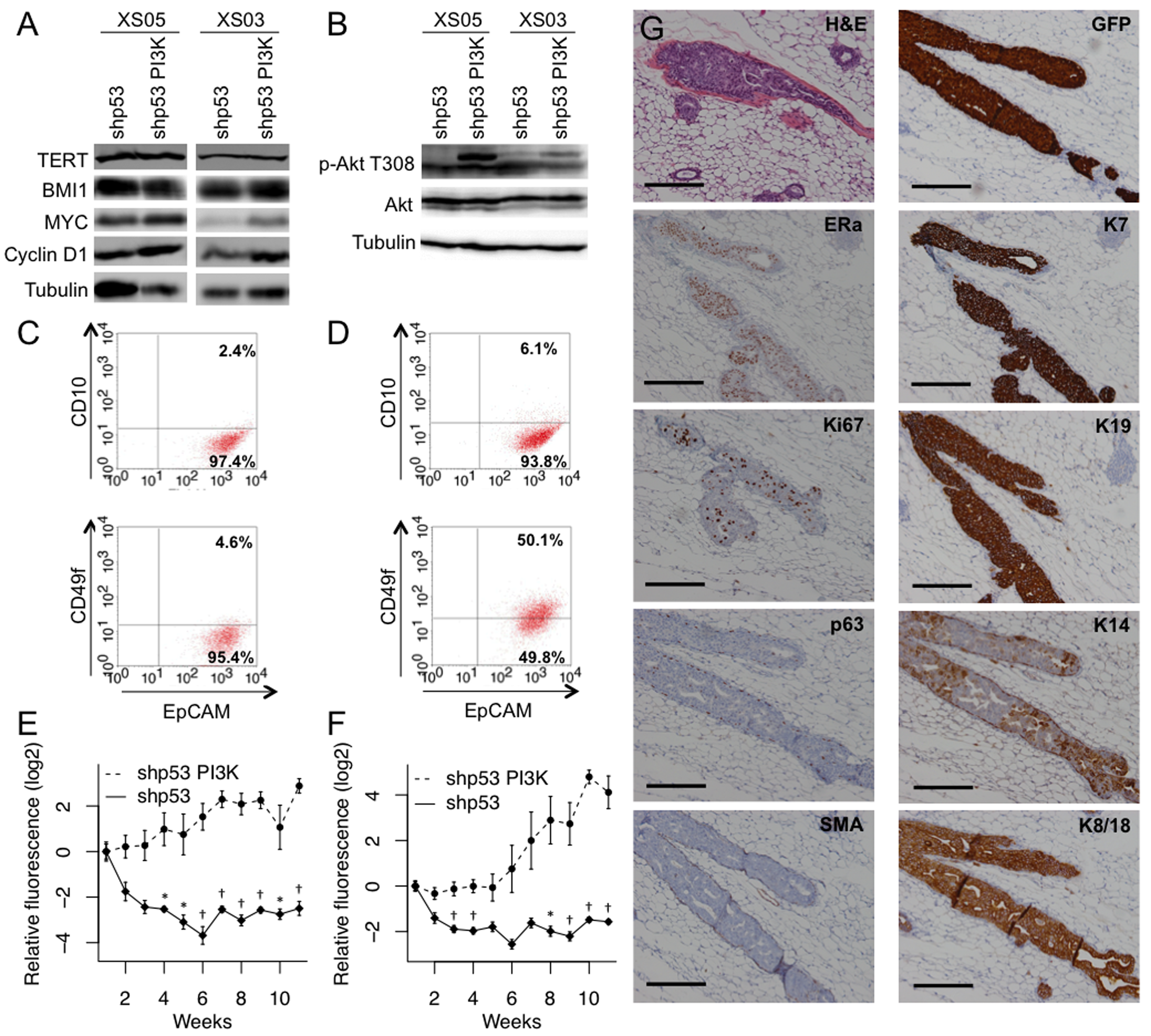

Supplement: Supplementary file 1 — Additional file 1: Figure S1: Transformation in svWIT with different mammoplasties (XS03 and XS05). A&B. Western blots showing expression of the TERT, BMI1, MYC and CCND1 transgenes (A) and phosphorylation of AKT on T308 induced by PI3K (B). C&D. Flow cytometry shows the formation of single populations of EPCAM+ cells after transformation (C, XS05; D, XS03). The quadrants were defined by the isotype controls. E&F. 4G-shp53 cells are unable to survive after subcutaneous xenografting. Only the cells superinfected with the PIK3CA vector survive and form tumors (E, XS05; F, XS03; n = 5). G. DCIS after intraductal injection of XS05 4G-shp53-PI3K cells (H&E staining and IHC for ERα Ki67, GFP, p63, SMA and keratins). Scale bars 100 μm. (TIFF 8310 kb) (TIFF 8 MB) [file 13058_2014_504_MOESM1_ESM.tiff]

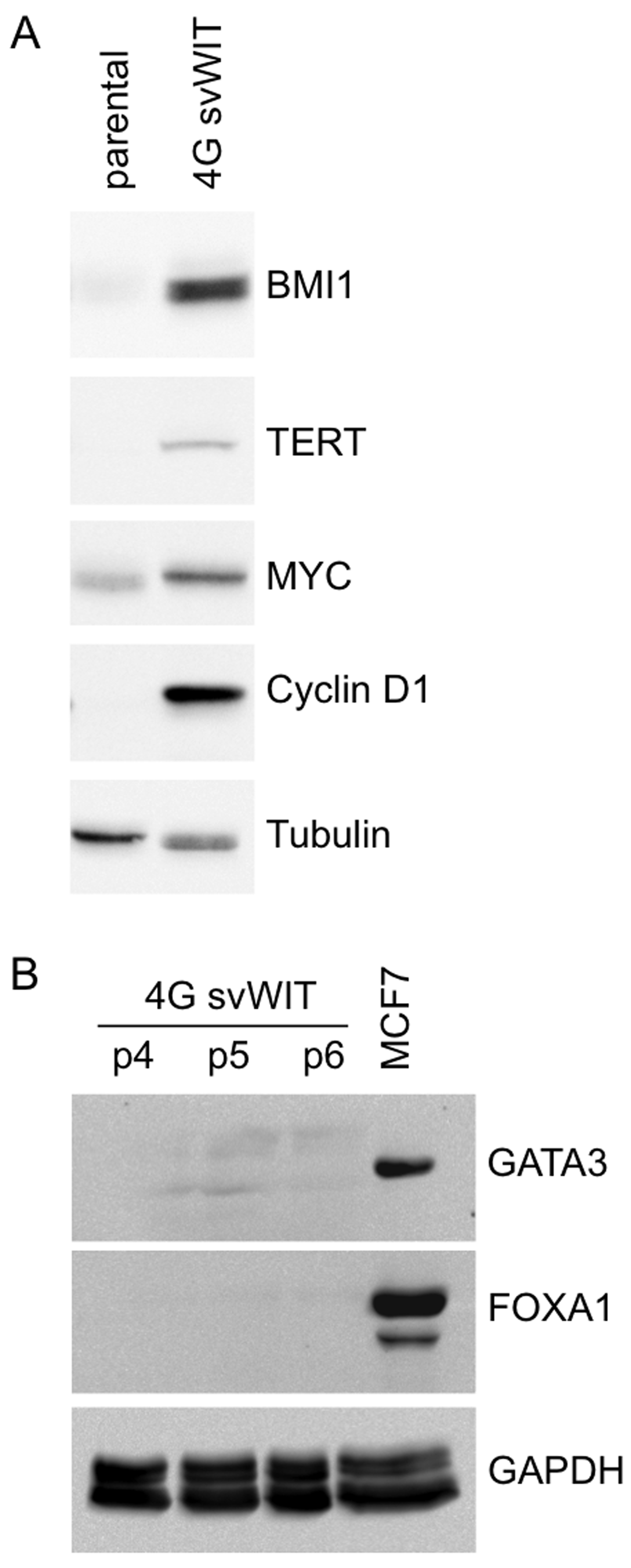

Supplement: Supplementary file 2 — Additional file 2: Figure S2: Western blots showing expression of the transgenes in 4G and parental cells (A) and lack of FOXA1 and GATA3 expression in 4G cells (B). MCF7 cells were used as a positive control for the FOXA1 and GATA3 blots. p4-p6, different passages of the 4G cells in svWIT medium. (TIFF 1460 kb) (TIFF 1 MB) [file 13058_2014_504_MOESM2_ESM.tiff]

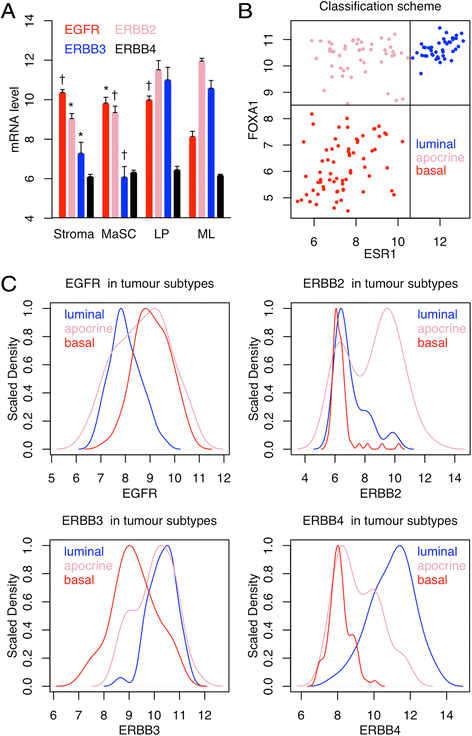

Supplement: Supplementary file 3 — Authors’ original file for figure 1 [file 13058_2014_504_MOESM3_ESM.gif]

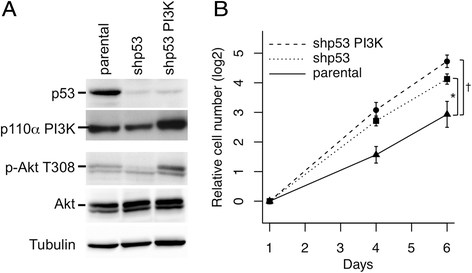

Supplement: Supplementary file 4 — Authors’ original file for figure 2 [file 13058_2014_504_MOESM4_ESM.gif]

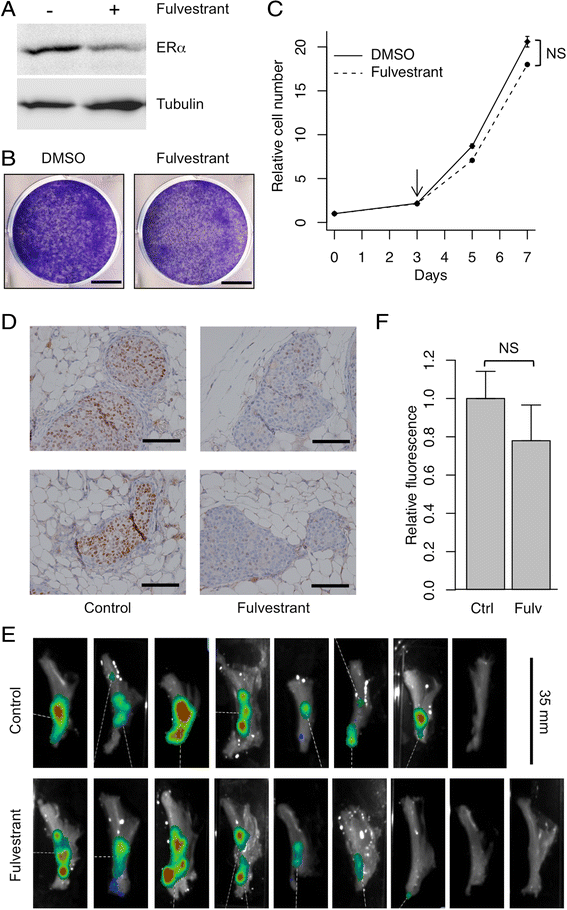

Supplement: Supplementary file 5 — Authors’ original file for figure 3 [file 13058_2014_504_MOESM5_ESM.gif]

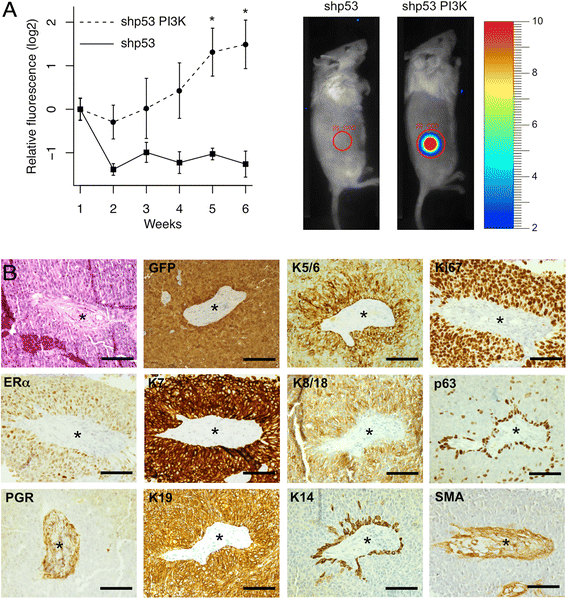

Supplement: Supplementary file 6 — Authors’ original file for figure 4 [file 13058_2014_504_MOESM6_ESM.gif]

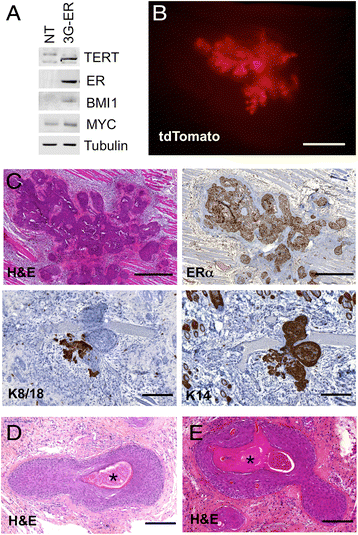

Supplement: Supplementary file 7 — Authors’ original file for figure 5 [file 13058_2014_504_MOESM7_ESM.gif]

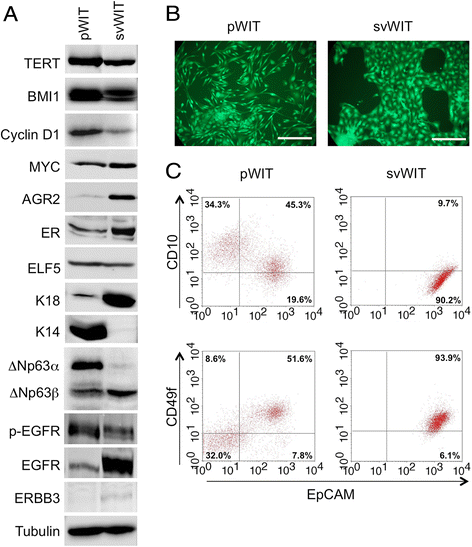

Supplement: Supplementary file 8 — Authors’ original file for figure 6 [file 13058_2014_504_MOESM8_ESM.gif]

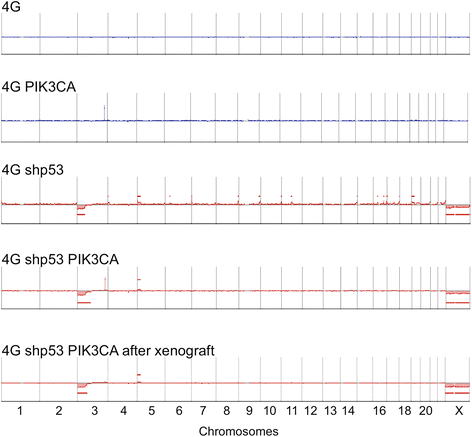

Supplement: Supplementary file 9 — Authors’ original file for figure 7 [file 13058_2014_504_MOESM9_ESM.gif]

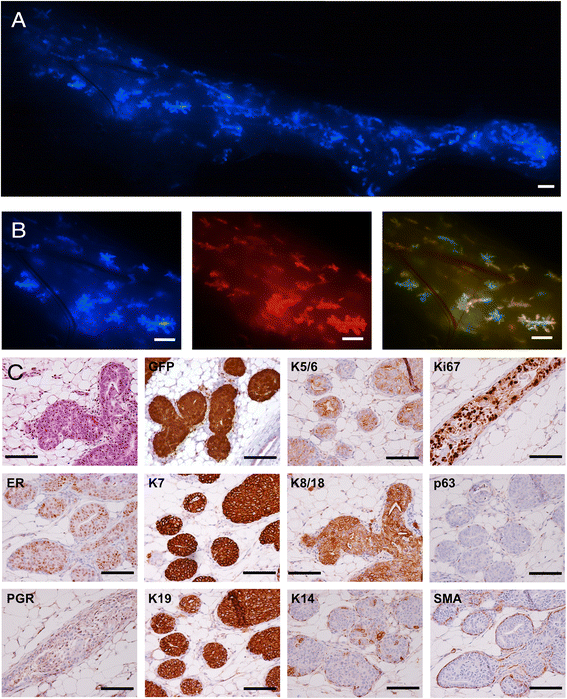

Supplement: Supplementary file 10 — Authors’ original file for figure 8 [file 13058_2014_504_MOESM10_ESM.gif]

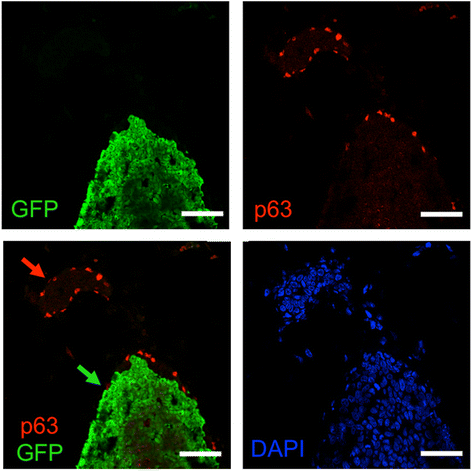

Supplement: Supplementary file 11 — Authors’ original file for figure 9 [file 13058_2014_504_MOESM11_ESM.gif]

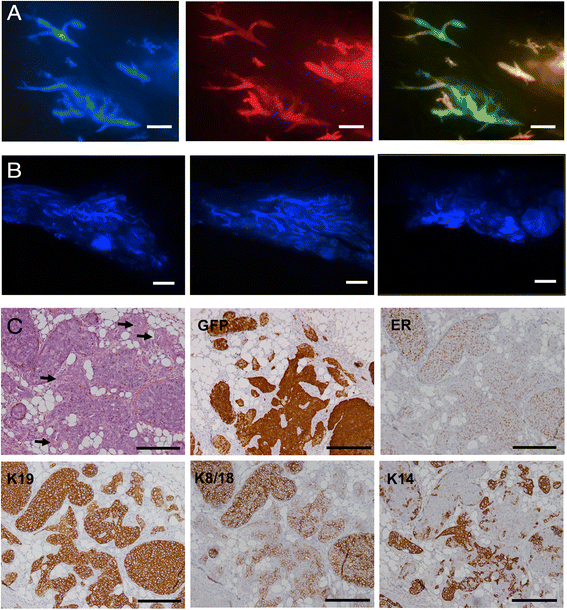

Supplement: Supplementary file 12 — Authors’ original file for figure 10 [file 13058_2014_504_MOESM12_ESM.gif]

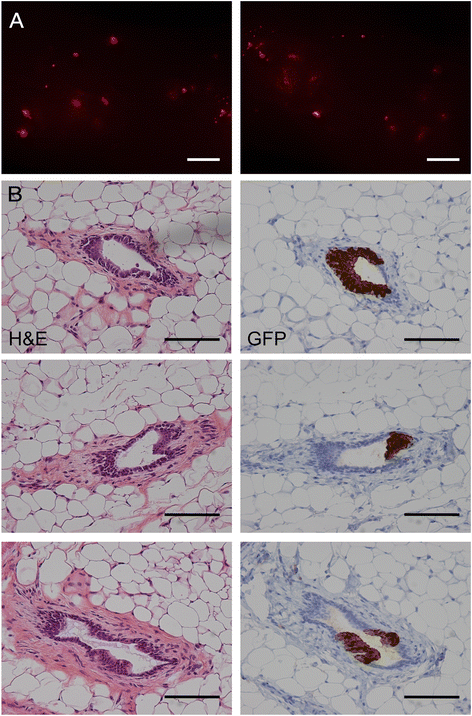

Supplement: Supplementary file 13 — Authors’ original file for figure 11 [file 13058_2014_504_MOESM13_ESM.gif]

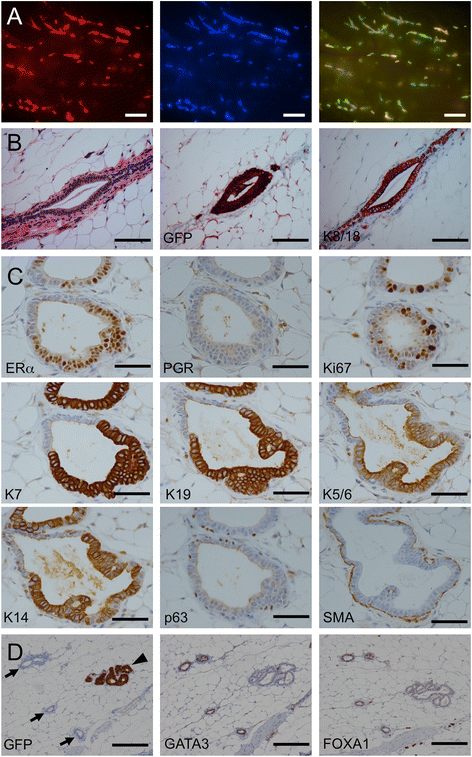

Supplement: Supplementary file 14 — Authors’ original file for figure 12 [file 13058_2014_504_MOESM14_ESM.gif]

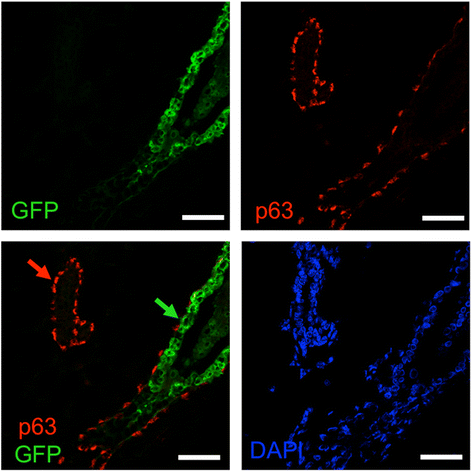

Supplement: Supplementary file 15 — Authors’ original file for figure 13 [file 13058_2014_504_MOESM15_ESM.gif]

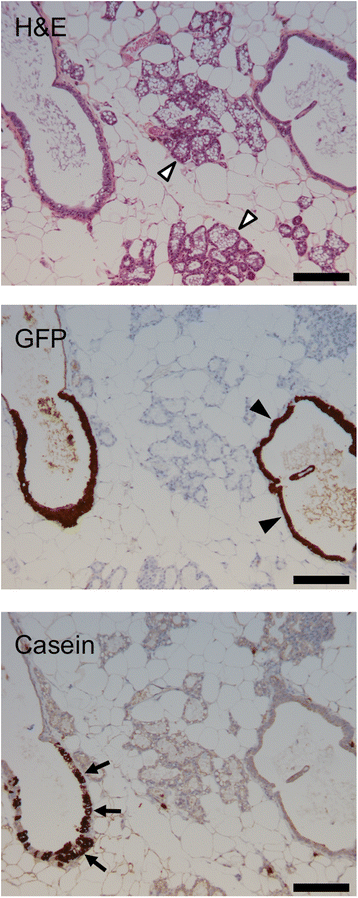

Supplement: Supplementary file 16 — Authors’ original file for figure 14 [file 13058_2014_504_MOESM16_ESM.gif]
